# Chromosomal microarray analysis of 410 Han Chinese patients with autism spectrum disorder or unexplained intellectual disability and developmental delay

**DOI:** 10.1038/s41525-021-00271-z

**Published:** 2022-01-12

**Authors:** Yi Liu, Yuqiang Lv, Mehdi Zarrei, Rui Dong, Xiaomeng Yang, Edward J. Higginbotham, Yue Li, Dongmei Zhao, Fengling Song, Yali Yang, Haiyan Zhang, Ying Wang, Stephen W. Scherer, Zhongtao Gai

**Affiliations:** 1 Pediatric Research Institute, Qilu Children’s Hospital of Shandong University, Ji’nan, 250022 China; 2 The Centre for Applied Genomics and Department of Genetics and Genome Biology, The Hospital for Sick Children, Toronto, ON M5G 0A4 Canada; 3 Pediatric Health Care Institute, Qilu Children’s Hospital of Shandong University, Ji’nan, 250022 China; 4 Rehabilitation Center, Qilu Children’s Hospital of Shandong University, Ji’nan, 250022 China; 5 McLaughlin Centre and Department of Molecular Genetics, University of Toronto, Toronto, ON M5S 1A1 Canada

**Keywords:** Neurodevelopmental disorders, Molecular medicine

## Abstract

Copy number variants (CNVs) are recognized as a crucial genetic cause of neurodevelopmental disorders (NDDs). Chromosomal microarray analysis (CMA), the first-tier diagnostic test for individuals with NDDs, has been utilized to detect CNVs in clinical practice, but most reports are still from populations of European ancestry. To contribute more worldwide clinical genomics data, we investigated the genetic etiology of 410 Han Chinese patients with NDDs (151 with autism and 259 with unexplained intellectual disability (ID) and developmental delay (DD)) using CMA (Affymetrix) after G-banding karyotyping. Among all the NDD patients, 109 (26.6%) carried clinically relevant CNVs or uniparental disomies (UPDs), and 8 (2.0%) had aneuploidies (6 with trisomy 21 syndrome, 1 with 47,XXY, 1 with 47,XYY). In total, we found 129 clinically relevant CNVs and UPDs, including 32 CNVs in 30 ASD patients, and 92 CNVs and 5 UPDs in 79 ID/DD cases. When excluding the eight patients with aneuploidies, the diagnostic yield of pathogenic and likely pathogenic CNVs and UPDs was 20.9% for all NDDs (84/402), 3.3% in ASD (5/151), and 31.5% in ID/DD (79/251). When aneuploidies were included, the diagnostic yield increased to 22.4% for all NDDs (92/410), and 33.6% for ID/DD (87/259). We identified a de novo CNV in 14.9% (60/402) of subjects with NDDs. Interestingly, a higher diagnostic yield was observed in females (31.3%, 40/128) compared to males (16.1%, 44/274) for all NDDs (*P* = 4.8 × 10^−4^), suggesting that a female protective mechanism exists for deleterious CNVs and UPDs.

## Introduction

Neurodevelopmental disorders (NDDs) are a group of heterogeneous disorders involving developmental dysfunction of the central nervous system, and have an incidence rate of 1–3% in children^1–3^. Autism spectrum disorder (ASD) and intellectual disability/developmental delay (ID/DD) are the most common NDDs in children and have shared psychiatric behaviors, clinical manifestations, and risk factors, and impair cognitive functions including learning, sociability, and mood^4^. ASD is one of the childhood-onset NDDs, characterized by impairment in three domains: social interaction, communication skills, and repetitive behavior and restricted interests. Approximately 50% of autistic cases manifest intellectual disability (ID)^5,6^. Developmental delay (DD) is the failure to achieve certain developmental milestones at the appropriate age, involving physical, cognitive, communication, social, emotional, and/or adaptive skills^3^. Both ID (IQ < 70) and DD belong to the clinically heterogeneous NDDs^3^.

The etiology of ASD and ID/DD is complex and overlapping, implicating both genetic and non-genetic factors. Chromosomal microarray analysis (CMA) is a molecular cytogenetic technique that expedites genome-wide detection of clinically significant copy number variants (CNVs), and has been recommended as a first-tier diagnostic tool for patients with ASD, unexplained ID/DD, and multiple congenital anomalies (MCA). CMA can accurately detect different types of CNVs (i.e., deletions or duplications) across a large size range (esp. <5 Mb submicroscopic CNVs), and can also identify uniparental disomies (UPDs)^7–9^. CMA has been demonstrated to improve the diagnostic yield to up to 33%, compared to 3.7% using karyotyping. However, the diagnostic yield can vary widely and largely depends on the cohort population and severity of phenotypes^9–11^.

CNV studies have been performed for patients with NDDs worldwide, but additional data from Chinese subjects are needed to delineate the potential differences in CNV distribution in this population, particularly within the clinical setting. Here, we present an investigation of CNVs in a well-characterized clinical cohort with ASD or ID/DD in Shandong, a province in northern China. CNVs were analyzed using the Affymetrix SNP 6.0 or CytoScan HD arrays in combination with deep mining databases, including an updated in-house database, to explore the clinical implication and inheritance of specific candidate CNVs or genes associated with NDDs.

## Results

### General diagnostic yield

Samples from 410 Han Chinese ASD and ID/DD patients were tested, of which 5 with highly suspected trisomy 21 syndrome were analyzed by G-banding karyotyping. Trisomy 21 was detected in these five samples. The remaining 405 samples were analyzed by array and three additional aneuploidies were found: one trisomy 21, one 47,XXY, and one 47,XYY. Thus, aneuploidies were identified in eight subjects (2.0%) in our cohort, with six trisomy 21, one 47,XXY, and one 47,XYY identified.

The remaining 402 cases (151 ASD and 251 ID/DD) were further analyzed for the presence of clinically relevant CNVs, which were defined as pathogenic CNVs, likely pathogenic CNVs, and CNVs interpreted as variants of uncertain significance (VUS). We detected 594 high-quality CNVs in our cohort (Supplementary Data 2), and selected 162 rare CNVs for experimental validation, of which 150/162 were confirmed as true calls. We interpreted 129 CNVs and uniparental disomies (UPDs) as clinically relevant, which were identified in 109 NDD cases (27.1%, 109/402). This included 32 CNVs in 30 ASD cases (19.9%), and 97 CNVs and UPDs in 79 ID/DD cases (31.5%). Four loci of mosaicism were found in our cohort: 9p24 duplication (x3–4), 15q11.2q13.3 duplication (x3–4), 18q21.31q23 deletion (x1–2), and 22q11.1q11.21 duplication (x3–4).

The 32 clinically relevant CNVs identified in 30 ASD cases included four pathogenic CNVs associated with known genetic syndromes, one likely pathogenic CNV, and 27 CNVs interpreted as variants of uncertain significant (VUS) according to ACMG classification (Table 2). There were 14 (43.8%) clinically relevant deletions and 18 (56.3%) clinically relevant duplications identified, of which five (15.6%) occurred de novo, 25 (78.1%) were inherited (16 paternal and 9 maternal), and two (6.3%) were of unknown inheritance (Tables 1 and 2, and Supplementary Data 3).

**Table 1:** Diagnostic yield of CNVs identified in the cohort.

Clinical diagnosis	Age (years)	Total no.	Sex, no.	Chromosomal syndrome (No.)	Pathogenic (No.)	Likely pathogenic (No.)	Diagnostic yield (%)
ASD	1–2	2	Male, 2	0	0	0	0
			Female, 0	0	0	0	0
	2–5	114	Male, 94	3	3	0	4.3(3/94)
			Female, 20	1	1	0	5.0(1/20)
	>5	35	Male, 31	0	0	1	2.9(1/35)
			Female, 4	0	0	0	0
	Total	151	Male, 127	3	3	1	3.1(4/127)
			Female, 24	1	1	0	4.2(1/24)
			M + F, 151	4	4	1	3.3(5/151)
ID/DD	<1	104	Male, 58	11	13	0	22.4(13/58)
			Female, 46	7	10	3	28.3(13/46)
	1–2	62	Male, 33	12	12	0	36.4(12/33)
			Female, 29	9	13	0	44.8(13/29)
	2–5	68	Male, 43	10	13	1	32.6(14/43)
			Female, 25	11	11	0	44.0(11/25)
	>5	17	Male, 13	0	1	0	7.7(1/13)
			Female, 4	2	2	0	50.0(2/4)
	Total	251	Male, 147	33	39	1	27.2(40/147)
			Female, 104	29	36	3	37.5(39/104)
			M + F, 251	62	75	4	31.5(79/251)
ASD + ID/DD	Total	402	Male, 274	35	42	2	16.1(44/274)
			Female, 128	29	37	3	31.3(40/128)
			M + F, 402	64	79	5	20.9(84/402)

**Table 2:** List of clinically relevant CNVs found in the patients with autism spectrum disorder.

Genes involved	No. of subjects	Cytoband	Size (kb)	CNV type	parent of origin	Genetic syndrome	Pathogenicity
*Pathogenic/likely pathogenic CNVs*							
*CADM2* + 10 genes	1	3p12.2p11.1	8418	Deletion	De novo	—	Likely pathogenic
*AUTS2*	1	7q11.22	830	Deletion	De novo	*AUTS2* syndrome	Pathogenic
*EHMT1*	1	9q34.3	801	Deletion	De novo	Kleefstra syndrome	Pathogenic
*BBS4*, *NEO1* + 31 genes	1	15q24.1q24.2	2581	Deletion	De novo	15q24 microdeletion syndrome	Pathogenic
*MECP2* + 15 genes	1	Xq28	408	Duplication	Maternal	*MECP2* microduplication syndrome	Pathogenic
*Variants of uncertain significance (VUS)*							
*CTNNA2* + 6 genes	1	2p12	1197	Duplication	Paternal	—	VUS
*DPP10*	1	2q14.1	141	Duplication	Paternal	—	VUS
*CNTNAP5*	1	2q14.3	477	Duplication	Maternal	—	VUS
*TRIP12*	1	2q36.3	115	Duplication	Maternal	—	VUS
*GRM7*	2	3p26.1	303–1174	Deletion	De novo (1), maternal (1)	—	VUS
*RSRC1*	1	3q25.32	435	Deletion	Paternal	—	VUS
*PIGG*	1	4p16.3	159	Deletion	Paternal	—	VUS
*ARHGEF38* + 3 genes	1	4q24	162	Duplication	Paternal	—	VUS
*KHDRBS2*	1	6q12	699	Duplication	Paternal	—	VUS
*AHI1*	1	6q23.3	103	Duplication	Paternal	—	VUS
*PARK2*	2	6q26	76–174	Deletion	Paternal (2)	—	VUS
*SDK1*	1	7p22.2	119	Duplication	Unknown	—	VUS
*GRM8*	1	7q31.33	56	Duplication	Paternal	—	VUS
*CTNNA3*	1	10q21.3	74	Deletion	Maternal	—	VUS
*LRRC4C*	1	11p12	208	Duplication	Paternal	—	VUS
*APBA2* + 5 genes	1	15q13.1	1715	Duplication	Paternal	—	VUS
*CHRNA7* + *OTUD7A*	1	15q13.3	435	Duplication	Paternal	—	VUS
*RBFOX1*	1	16p13.3	294	Deletion	Paternal	—	VUS
*GRIN2A*	1	16p13.2	452	Duplication	Paternal	—	VUS
*LAMA1* + 2 genes	1	18p11.23p11.31	517	Duplication	Unknown	—	VUS
*MIB1*	1	18q11.2	122	Deletion	Maternal	—	VUS
*PTPRT*	1	20q12	77	Deletion	Maternal	—	VUS
*AIFM3*	1	22q11.21	412	Duplication	Paternal	—	VUS
*IGBP1* + 10 genes	1	Xq13.1	401	Duplication	Maternal	—	VUS
*SMS* + *PHEX*	1	Xq22.11	75	Duplication	Maternal	—	VUS

In contrast, the 97 CNVs and UPDs in ID/DD cases included 90 pathogenic variants (72 implicating 34 known genetic syndromes), four likely pathogenic variants, and three VUS based on ACMG classification (Tables 1 and 3). The clinically relevant CNVs and UPDs included 53 (54.6%) deletions, 39 (40.2%) duplications, and five (5.2%) UPDs. The majority of CNVs (67.01%, 65/97) were de novo, and the proportion of ID/DD subjects with a de novo CNV was 21.9% (55/251), much higher than that of ASD subjects (*P* = 1.0 × 10^−7^). We found that 12.4% of the CNVs and UPDs were inherited (3 paternal and 9 maternal), and 20.6% were of unknown inheritance due to the unavailability of parental samples (Table 1 and 3, and Supplementary Data 4). The inheritance of the five UPDs was determined, revealing two cases with Prader–Willi syndrome, one case with Angelman syndrome, one case with Silver–Russell syndrome, and one case with maternal UPD in 14q.

**Table 3:** Clinically relevant CNVs found in the patients with intellectual disability and developmental delay.

Region name	No. of subjects	Cytoband	CNV type	Size (kb)	Parent of origin	Pathogenicity
*Genetic syndromes*						
1p36 deletion syndrome	2	1p36.33p36.23; 1p36.33p36.32	Deletion	4515–7908	De novo (2)	Pathogenic
1q21.1 microduplication syndrome	1	1q21.1q21.2	Duplication	1626	Maternal	Pathogenic
2q37 microdeletion syndrome	2	2q37.3	Deletion	3656–3973	De novo (2)	Pathogenic
Wolf-Hirschhorn syndrome	1	4p16.3p16.2	Deletion	5868	De novo	Pathogenic
Cri-du-chat syndrome	2	5p15.33p15.1; 5p15.31p15.1	Deletion	7595–16,576	De novo (2)	Pathogenic
5q trisomy syndrome	1	5q34q35.3	Duplication	15,248	De novo	Pathogenic
Sotos syndrome	1	5q35.2q35.3	Deletion	1911	De novo	Pathogenic
Silver–Russell syndrome	1	7p13q34	UPD	95,119	maternal	Pathogenic
Williams–Beuren syndrome	11	7q11.23	Deletion	1424–1570	De novo (11)	Pathogenic
Waardenburg syndrome	3	8p23.3p23.1	Deletion	6841–9905	De novo (1), unknown (2)	Pathogenic
Branchio–Otorenal syndrome	1	8q12.3q13.3	Deletion	10,009	De novo	Pathogenic
9 partial monosomy syndrome	1	9p24.3p23	Deletion	10,487	De novo	Pathogenic
9p partial trisomy	5	9p24.3p13.1; 9p24.3p21.1; 9p24.3q21.11; 9p24.3p21.13	Duplication (4), duplication x3 + duplication x4 (1)^a^	29,995–74,596	De novo (5)	Pathogenic
10p partial deletion	1	10p15.3p14	Deletion	11,049	De novo	Pathogenic
10p15.3 deletion syndrome	1	10p15.3p15.2	Deletion	3472	De novo	Pathogenic
Distal trisomy 10q syndrome	1	10q24.2q26.3	Duplication	35,938	De novo	Pathogenic
10qter deletion syndrome	1	10q26.2q26.3	Deletion	5640	De novo	Pathogenic
Prader–Willi syndrome (deletion)	2	15q11.2q13.1	Deletion	5259–6261	Paternal (2)	Pathogenic
Prader–Willi syndrome (UPD)	2	15q11.2q26.3	UPD (2)	79,659–79,677	Maternal	Pathogenic
Angelman syndrome (deletion)	1	15q11.2q13.1	Deletion	5239	Maternal	Pathogenic
Angelman syndrome (UPD)	1	15q11.2q21.1	UPD	25,942	Paternal	Pathogenic
15q11-q13 duplication syndrome	4	15q11.2q13.1; 15q11.2q13.2; 15q13.2q13.3	Duplication (1), duplication x3 (1), duplication x4 (1), duplication x3 + duplication x4 (1)^b^	4929–9674	De novo (3), unknown (1)	Pathogenic
15q26 deletion syndrome	1	15q26.3	Deletion	3134	Unknown	Pathogenic
16p13.11 microduplication syndrome	1	16p13.11	Duplication	1429	maternal	VUS
16p11.2 microduplication syndrome (BP4-BP5)	2	16p11.2	Duplication	586–826	De novo (1), unknown (1)	Pathogenic
16 partial trisomy syndrome	1	16q22.1q24.3	Duplication	19,668	De novo	Pathogenic
17 partial trisomy syndrome	1	17q25.3	Duplication	5701	De novo (1)	Pathogenic
18q deletion syndrome	4	18q21.2q21.31; 18q21.31q23; 18q21.33q23; 18q22.3q23	Deletion (3), deletion x1–2 (1)	3042–77,943	De novo (3), unknown (1)	Pathogenic
18q duplication syndrome	1	18q21.32q23	Duplication	77,943	De novo	Pathogenic
22q11.2 duplication syndrome	1	22q11.1q11.21; 22q11.21	Duplication x4 + duplication x3^c^	2893; 1682	De novo	Pathogenic
22q11.2 deletion syndrome	3	22q11.21	Deletion	2549–2884	De novo (3)	Pathogenic
*MECP2* microduplication syndrome	2	Xq28	Duplication	377–424	Maternal (1), paternal (1)	Pathogenic
*Other clinically relevant CNVs*						
1q25.1q31.1 deletion	1	1q25.1q31.1	Deletion	12,292	De novo	Pathogenic
2p16.1p12 deletion	1	2p16.1p12	Deletion	16,664	Unknown	Pathogenic
2p21p13.2 duplication	1	2p21p13.2	Duplication	28,560	De novo	Pathogenic
2p25.3p25.2 duplication	1	2p25.3p25.2	Duplication	6544	Unknown	Pathogenic
2q13 deletion	1	2q13	Deletion	1734	De novo	Likely pathogenic
3p14.1p11.1 duplication	1	3p14.1p11.1	Duplication	21,577	De novo	Pathogenic
4p16.3p16.1 duplication	1	4p16.3p16.1	Duplication	9452	Unknown	Pathogenic
4p16.3 deletion	1	4p16.3	Deletion	1195	Unknown	Pathogenic
4p16.2p16.1 duplication	1	4p16.2p16.1	Duplication	493	De novo	VUS
4q31.3q35.2 duplication	1	4q31.3q35.2	Duplication	38,587	Unknown	Pathogenic
4q34.14q35.2 duplication	1	4q34.14q35.2	Duplication	18,897	De novo	Pathogenic
6q23.3q24.2 deletion	1	6q23.3q24.2	Deletion	7405	Unknown	Pathogenic
7q11.22q21.11 deletion	1	7q11.22q21.11	Deletion	17,988	De novo	Likely pathogenic
8p23.3p22 deletion	1	8p23.3p22	Deletion	17,300	De novo	Pathogenic
13q21.1q32.2 deletion	1	13q21.1q32.2	Deletion	39,073	De novo	Pathogenic
14q11.2q21.2 duplication	1	14q11.2q21.2	Duplication	23,595	Unknown	Pathogenic
14q11.2q32.33 UPD	1	14q11.2q32.33	UPD	86,774	maternal	Pathogenic
14q32.12p32.33 duplication	1	14q32.12p32.33	Duplication	11,616	Unknown	Pathogenic
15q26.2q26.3 deletion	1	15q26.2q26.3	Deletion	3006	De novo	Pathogenic
16q24.2q24.3 duplication	1	16q24.2q24.3	Duplication	2671	Unknown	Pathogenic
18p11.32p11.31 deletion	1	18p11.32p11.31	Deletion	5987	De novo	Pathogenic
18p11.32p11.21 deletion	1	18p11.32p11.21	Deletion	13,649	De novo	Pathogenic
18q21.32q23 duplication	1	18q21.32q23	Duplication	19,416	De novo	Pathogenic
19p13.2 deletion	1	19p13.2	Deletion	2260	De novo	Likely pathogenic
19p13.3p13.2 duplication	1	19p13.3p13.2	Duplication	7327	De novo	Pathogenic
20p13 deletion	1	20p13	Deletion	978	De novo	Pathogenic
20p13p12.3 deletion	1	20p13p12.3	Deletion	9844	Unknown	Pathogenic
21q22.11q22.3 deletion	1	21q22.11q22.3	Deletion	13,055	De novo	Pathogenic
21q22.3 duplication	1	21q22.3	Duplication	2858	De novo	VUS
22q13.31q13.33 duplication	2	22q13.31q13.33	Duplication	5648	Unknown (2)	Pathogenic
Xq22.3q27.1 duplication	1	Xq22.3q27.1	Duplication	32,324	Unknown	Pathogenic

*UPD* uniparental disomy, *VUS* variant of uncertain significance.

^a^This subject has a de novo duplication (x4) of chr9:203,861–4,199,819, immediately adjacent to a de novo duplication (x3) of chr9:4,199,820–38,787,479.

^b^This subject has a duplication (x4) of chr15:22,770,421–31,073,668, immediately adjacent to a duplication (x3) of chr15:31,073,668–32,011,459.

^c^This subject has a de novo duplication (x4) of chr22:16,888,899–19,781,868, immediately adjacent to a de novo duplication (x3) of chr22:19,783,504–21,465,659.

To obtain a more stringent estimate of the diagnostic yield for our cohort, we considered only subjects with pathogenic/likely pathogenic CNVs and UPDs in the calculation. The diagnostic yield was 20.9% (84/402) for all NDDs, and increased to 22.4% (92/410) when subjects with aneuploidies were included. The diagnostic yield was markedly lower for ASD (5/151, 3.3%) compared to ID/DD (79/251, 31.5% without aneuploidies; 87/259, 33.6% including aneuploidies).

The size range of the clinically relevant CNVs and UPDs was 56 kb to 95.1 Mb in the entire cohort, 56 kb to 8.4 Mb in subgroup of ASD cases, and 377 kb to 95.1 Mb in the subgroup of ID/DD cases. The average size of positive CNVs and UPDs was 802.5 kb ± 394.5 kb in ASD cases compared to 4.14 Mb ± 3.77 Mb in ID/DD cases, which showed a significant difference between the two subgroups (*P* = 0.005). We then compared the average size of CNVs and UPDs identified in males and females of both subgroups. There was no statistical difference in the average size of CNVs identified in males and females in the ASD subgroup (802.5 kb ± 394.5 kb in males compared to 316 kb ± 201 kb in females). In contrast, the average size of CNVs and UPDs was 2.45 Mb ± 2.07 Mb in males and 20.12 Mb ± 12.21 Mb in females in the ID/DD subgroup, indicating a significant difference between male and female ID/DD cases (*P* = 0.001). The majority of clinically relevant CNVs and UPDs (95/129, 73.6%) were smaller than 10 Mb in size (i.e., submicroscopic) and would not be identified by karyotyping (Tables 2 and 3 and Supplementary Data 4).

### CNVs in regions of known chromosomal syndromes

Seventy-six CNVs were identified at regions associated with 37 well-known chromosomal microdeletion/microduplication syndromes. Four pathogenic CNVs associated with known genetic syndromes were identified in four ASD cases. A 4-year-old boy with ASD and DD harbored an 830 kb deletion at 7q11.22 that overlapped the *AUTS2* gene. He was diagnosed with *AUTS2* syndrome. A 3-year-6-month girl with ASD, DD and congenital heart disease harbored a 9q34.3 deletion and was diagnosed with Kleefstra syndrome. A 2-year-6-month boy with ASD and DD harbored a 2.58 Mb deletion at chr15q24, and was considered to have 15q24 microdeletion syndrome. Last, a 3-year-3-month male patient harbored a 408 kb duplication at chrXq28 including *MECP2*, and was diagnosed with *MECP2* duplication syndrome (Table 2 and Supplementary Data 3).

There were 73 CNVs and four UPDs identified in 60 ID/DD cases that were associated with known chromosomal syndromes. Williams–Beuren syndrome deletions were the most commonly observed syndromic CNV, and were identified in 11 cases (Table 3). Examples of other CNVs and UPDs identified in multiple cases with ID/DD included five partial trisomy 9p, four 15q11q13 duplication syndrome, four 18q deletion syndrome, four Prader–Willi syndrome (2 paternal deletions and 2 maternal UPDs), and three 22q11 deletion syndrome, among others (Table 3 and Supplementary Data 4).

The patients with known chromosomal syndromes presented with heterogeneous clinical features, including three cases with de novo deletions of the 22q11.2 deletion syndrome region. The first 22q11.2 deletion syndrome case, 15D1529, was a 23-month-15-day-old boy, the first child of non-consanguineous healthy parents. His clinical features included special appearance such as sparse hairs, small eyes, low-ears, thick lips, and irregular teeth. He had a height of 83.5 cm, weight of 10 kg, and head circumference of 45.1 cm. His speech was delayed significantly with only babbling. His development was assessed using the Gesell Developmental Observation-Revised (GDO-R), and his motor, social, and language development were all found to be delayed. His blood biochemical tests, head MRI and heart ultrasound were normal. The second case, 15D3173, was an 8-day-old boy, who was the third child of non-consanguineous healthy parents, and had two healthy sisters of 10 years old and 7 years old, respectively. He was referred to the hospital because of seizures and abnormal development, and presented with facial dysmorphism, specifically small mouth, micrognathia, and high arched palate. His growth delay in utero was found before he was born, and physical examination and ultrasonic cardiogram demonstrated that he had atrioventricular septal defect (AVSD), and pulmonary arterial hypertension. His blood tests showed he had hypocalcemia with calcium 1.38 mmol/L and immunodeficiency with IgA 0.04 g/L (normal: 0.03–0.82 g/L), IgG 8.37 g/L (normal: 7.00–14.40 g/L), IgM 0.052 g/L (normal: 0.06–0.20 g/L). His cryptorchidism and polydactyl were also noticed. The third case, 19D0970, was a 6-day-old girl and the first child of non-consanguineous healthy parents. She was referred to the hospital for seizures and fever, and presented with facial dysmorphism, with features that included small jaw, flat bridge of nose, narrow nasal passages, laryngeal dysplasia with softening of the laryngeal cartilage, and softening of the trachea. She also had growth delay in utero before her birth. Ultrasound examination showed she suffered from congenital heart defects of patent ductus arteriosus, pulmonary hypertension, tricuspid regurgitation, and patent foramen ovala as well as smaller thymus (1.7*1.5*0.6 cm). Her blood tests showed she had lower calcium (0.83 mmol/L, normal: 2.2–3.0 mmol/L) and congenital hypothyroidism with TSH 15.22 mIU/L (normal: 0.98–5.63 mIU/L) and FT4 2.53 pmol/L (normal: 11.4–19.5 pmol/L).

### De novo variants

We identified 70 de novo CNVs in 60 patients with NDDs. This included 65 de novo CNVs identified in 55 ID/DD cases (31 males and 24 females) and five de novo CNVs identified in five ASD cases (4 males and 1 female). The proportion of subjects with de novo CNVs was 14.9% (60/402) for all 402 NDD patients (excludes patients with aneuploidies), 3.3% (5/151) for ASD cases and 21.9% (55/251) for the ID/DD cases. The proportion of subjects with a de novo CNVs was significantly higher in the ID/DD subgroup compared to the ASD subgroup (*P* = 1.0 × 10^−7^) (Tables 2 and 3, and Supplementary Data 3 & 4).

Of the 70 de novo CNVs found in NDDs, 75.7% (53/70) occurred at loci associated with known chromosomal syndromes, including three CNVs identified in ASD cases and 50 CNVs identified in ID/DD cases. The three syndromic de novo CNVs in ASD subjects included *AUTS2* syndrome deletion, Kleefstra syndrome deletion, and 15q24 microdeletion syndrome (Table 2). The remaining two de novo CNVs identified in two ASD cases were an 8.4 Mb deletion at 3p12.2p11.1 and a 303 kb deletion at 3p26.1. The de novo deletion at 3p12.2p11.1 was detected in a 5-year-6-month boy who was diagnosed with severe autism. The deletion overlapped several genes that are highly expressed in the brain and might contribute to the phenotype, such as *CADM2*, *CHMP2B*, *POU1F1*, and *CGGBP1*. The other de novo deletion at 3p26.1 was found in a 4-year-old boy, and overlapped *GRM7*, the gene of metabotropic glutamate receptor 7 that is an emerging candidate gene for ASD and other neuropsychiatric disorders^12^^,13^.

Of the 65 de novo CNVs found in ID/DD cases, 50 were associated with known chromosomal syndromes (Table 3). Notably, the deletions of 7q11.23 Williams–Beuren syndrome region occurred de novo in all 11 patients, who were diagnosed with Williams–Beuren syndrome. Other examples of de novo CNVs associated with genetic syndromes included five de novo duplications of 9p24 (partial trisomy 5p), three de novo duplications of 15q11–13, and two subjects de novo deletions of 2q37. There were 15 de novo CNVs that occurred at regions that were not associated with known chromosomal syndromes. These included 10 pathogenic CNVs, three likely pathogenic CNVs, and two CNVs classified as VUS based on ACMG guideline (Table 3 and Supplementary Data 4).

### Cases with multiple clinically relevant CNVs

There were 20 cases who harbored two clinically relevant CNVs, of which two were ASD cases and 18 were ID/DD cases (Supplementary Data 3 and 4). The first ASD case (F3) was a 2-year-4-month-old girl who harbored a 115 kb maternally transmitted duplication overlapping *TRIP12* and a 74 kb maternally transmitted deletion overlapping *CTNNA3*, both of which were interpreted as VUS. The second ASD case (Y26) was a 3-year-10-month-old girl who harbored two CNVs interpreted as VUS: a 699 kb paternally transmitted duplication overlapping *KHDRBS2* and a 76 kb paternally transmitted deletion overlapping *PARK2*. The 18 ID/DD cases included 15 cases with two pathogenic (or likely pathogenic) CNVs, and three subjects with one pathogenic CNV and a second CNV interpreted as VUS. For example, a 1-year-old girl (16D1511) with DD and facial abnormalities harbored both a 6.5 Mb duplication at 2p25.3p25.2 and a 23.6 Mb duplication at chr14q11.2-q21.2. A 1-month-22-day-old girl (19D0185) with DD, bilateral hearing problem, valgus feet, and cleft palate had both a 17.38 Mb deletion at chr18q21.33q23 and a 9.84 Mb deletion at chr20p13p12.3. A 9.45 Mb duplication at chr4p16.3p16.1 and a 6.84 Mb deletion at chr8p23.3p23.1 were detected in a 19-month-8-day-old boy (19D1091) with DD, speech delay, and hearing problem. We found two cases with Williams–Beuren syndrome deletions who harbored a second clinically relevant CNV. Case (16D2191) harbored a de novo Williams–Beuren syndrome deletion and carried an additional pathogenic CNV, a 5.65 Mb duplication at 22q13.31q13.33. The second case (19D0262) harbored a Williams–Beuren syndrome deletion and a recurrent 1.4 Mb duplication at 16p13.11, which was interpreted as VUS^14^.

### New CNV candidates potentially related to ASD

In addition to the five pathogenic and likely pathogenic CNVs identified in the ASD subgroup, we identified 27 additional VUS in these cases (Table 2 and Supplementary Data 3). The VUS included 10 deletions and 17 duplications. One CNV occurred de novo, 24 were inherited, and two were of unknown inheritance. Analysis of data from the literature and disease databases suggests that many of these VUS are novel candidate CNVs for ASD and involve genes related to ASD/DD. Examples include a 303 kb de novo deletion and a 1.1 Mb maternal deletion at 3p26.1 that both overlap *GRM7*; a 141 kb paternal duplication at chr2q14.1 overlapping *DPP10*; a 477 kb maternal duplication at chr2q14.3 overlapping *CNTNAP5*; a 115 kb maternal duplication at chr2q36.3 overlapping *TRIP12*; a 435 kb paternal deletion at chr3q25.32 overlapping *RSRC1*; a 56 kb paternal duplication at chr7q31.33 overlapping *GRM8*; and a 452 kb paternal duplication at chr16p13.2 overlapping *GRIN2A*, among others (Supplementary Data 3).

## Discussion

The diagnostic yield of CMA varies across NDDs, with a higher detection rate of CNVs in patients with ID/DD than those with simplex ASD^15–22^. CMA has been applied for Chinese patients with NDDs in recent years, but the potential differences in CNV distribution in a clinical setting in China is not well illuminated. Here, we investigated the genetic etiology of 410 patients with ASD or ID/DD who were referred to our institute for clinical service by first using G-banding karyotyping and then genotyping samples using the Affymetrix SNP array 6.0 or CytoScan HD. Both platforms have high resolution and are capable of reliably detecting chromosomal structural abnormalities over 50 kb in size.

Pathogenic and likely pathogenic CNVs were detected in 84 subjects with NDDs (5 ASD and 79 ID/DD). Thus, the overall diagnostic yield was 20.9% in our cohort. However, it was significantly lower (3.3%) in subjects with ASD, while remarkably higher (31.6%) for subjects with ID/DD. We identified a de novo CNV in 14.9% of subjects with NDDs. We also found 20 ID/DD cases with more severe phenotypes who harbored two clinically relevant CNVs.

The diagnostic yield and de novo CNV rate of our cohort is comparable with some previous reports (Table 4). Our results are most consistent with Hu et al.^20^, who identified 127 cases carrying pathogenic CNVs in a cohort of 633 patients, obtaining a diagnostic yield of 20.06% for all NDD patients, 3.7% for isolated ASD, 18.07% for isolated ID/DD, and 34.90% for ID/DD with MCA. The size of CNVs identified in Hu et al. ranged from 223 kb to 102 Mb, and the de novo rate was 16.9%. Lee et al.^22^ obtained a similar diagnostic yield of 32.2% in 177 patients with unexplained ID/DD. Fan et al.^23^ observed a yield of 28% in a mixed cohort of 710 Southern Chinese patients with NDDs. The highest yield was found in the subgroup of ID/DD with congenital heart defects (55%), followed by ID/DD with facial dysmorphism (39%), hypotonia (35%), and microcephaly (34%). Pinto et al.^24^ obtained a diagnostic yield of 3.4% in a cohort of 2446 subjects with ASD and a de novo rate of 4.7%, but this was a strictly research cohort. This is comparable to our diagnostic yield of 2.6% in ASD subjects and de novo rate 3.3%. Ho et al.^18^ tested 10,351 NDDs cases and observed a yield of 5.4% for ASD/or combining with any other testing indications, which was also comparable to our ASD data. While their yield of 12.5% for subjects with ID/DD and 8.6% for all NDDs was lower than that observed in our study, it is comparable to some published data^8,25^. For example, Zarrei et al.^8^ obtained a yield of 10.5% in 1838 NDDs cases. A higher yield of 11.4% was observed in the ASD cases in this study. Uddin et al.^25^ also obtained a lower yield of 10.15% in a cohort of 10,619 subjects with NDDs. We suppose that the difference resulted from the constitution of cases with NDDs, which can include subjects with ASD, ADHD (attention deficit hyperactivity disorder), OCD (obsessive-compulsive disorder), and SCZ (schizophrenia) without many comorbid constructive defects. Our data further confirm that diagnostic yield is related positively with severities and comorbid conditions of NDDs, such as co-occurring facial dysmorphism and congenital heart diseases which could increase the yield markedly^18,21^.

**Table 4:** Comparison of diagnostic yield CNVs with different studies.

Total number of cases	CNV size (kb)	De novo rate	Yield			Source [reference no.]
			ASD	ID/DD	NDD	
402	56–95,100	17.4%	2.6%	31.1%	20.4%	This study
1838	10–155,271	5.6%	11.4%	NA	10.5%	Zarrei et al.^8^
10,351	NA	NA	5.4%	12.5%	8.6%	Ho et al.^18^
633	223–102,000	16.9%	3.7%	18.07–34.9%	20.06%	Hu et al.^20^
177	?−33,830	NA	NA	32.2%	32.2%	Lee et al.^22^
710	69–15,500	NA	NA	24–55%	28%	Fan et al.^23^
2446	30–59,374	4.7%	3.4%	NA	3.4%	Pinto et al.^24^
10,619	30–5000	NA	NA	NA	10.15%	Uddin et al.^25^

The diagnostic yield of a cohort can be affected significantly by multiple factors that include but are not limited to: referring physician specialty, gender of patients, age of patient at testing, and referring indication (or combination of indications) for testing^17^. The specialties of the referring pediatricians who are responsible for selecting patients to undergo genetic testing constitutes the first bias to the diagnostic yield in a clinical setting^17–19^. In this study, a diagnostic yield of 31.5% was obtained for the patients with ID/DD presenting clear clinical features, some of whom suffered from more severe and complex phenotypes. These patients were mostly (96%) diagnosed by senior developmental pediatricians or pediatric neurologists at the Pediatric Health Care Institute and Rehabilitation Center, and a few (4%) by senior and experienced neonatologists at the Neonatology Department in our hospital. Their specialty and experiences in recognizing the patients’ indications and severities is crucial to elevating the yield when referring them for genetic testing.

It is documented that the CNV burden differs between males and females in both NDD patients and the general population^26–32^. In our cohort, a higher diagnostic yield of pathogenic and likely pathogenic CNVs was observed in females (31.3%) compared to males (16.1%) when considering all NDDs, indicating a significant difference (*P* = 4.8 × 10^−4^). Similarly, in the ID/DD subgroup, a higher yield of 37.5% was observed in females compared to that of 27.2% in males, though this difference was not statistically significant. Jacquemont et al.^26^ investigated the molecular basis of the sex-based difference in a cohort of 15,585 probands with NDD, and found a significant increase in deleterious autosomal CNVs and single-nucleotide variants (SNVs) in female probands compared to males with NDDs. Jacquemont et al. also found that maternal transmission of deleterious CNVs and SNVs was observed more frequently in females than in males in an independent ASD cohort of 762 families. These data support the “female protective model”, suggesting a higher “mutational burden” is required for females with NDDs to manifest clinical features. In our study, the CNV size in females was larger than that in males in the ID/DD subgroup (20.12 Mb ± 12.21 Mb versus 2.45 Mb ± 2.07 Mb), demonstrating a significant difference between male and female patients (*P* = 1 × 10^−3^). This result is comparable to previous studies^25,26^. For example, Polyak et al.^27^ discovered that girls carried a higher burden of large CNVs and in both ASD and ID/DD cohorts. Han et al.^28^ found a significant excess of large (≥500 kb), rare (<1%) CNVs in females compared to males in both NDD cases and controls. Desachy et al.^29^ observed a similar phenomenon of large, rare CNVs in females in the population and ASD families, suggesting a female protective mechanism exists for deleterious CNVs that may go beyond NDDs phenotypes and contribute to decreased female fetal loss in the population. Roberts et al.^32^ observed a higher detection rate of abnormal CNVs in females (27%) than in males (18%). Roberts et al. also found that the average size of CNVs in ID/DD was much larger than that in ASD (2.90 ± 2.87 Mb versus 966 ± 1464 kb). Consistently, in our cohort, the average size of CNVs in ID/DD cases was 4.14 Mb ± 3.77 Mb, which was much larger than that of ASD cases (802.5 kb ± 394.5 kb). The difference is potentially related to the genetic cause of ID/DD and ASD, with smaller CNVs implicating a single gene in ASD versus larger CNVs involving more than one gene in ID/DD.

It has been noticed that the age of patients also affects the detection rate of CNVs^18,21^. Xu et al.^33^ investigated CNVs identified in 434 patients with ASD and ID/DD. The yield for patients under 2-year-old was 70%, which was significantly higher than those over 5 years old. Most of the younger patients under 2-year-old suffered from comorbidity with severe medical problems, such as microcephaly, macrocephaly, hypotonia, and other systemic abnormalities like asphyxia of the newborn, malnutrition and anemia, which may have influenced the pediatricians to recommend genetic evaluation for these patients with CMA. Moreover, similar yields of 12% and 14.7% were obtained for both subgroups of ASD and ID/DD under 2-year-old in this study. In order to illustrate the impact of patients’ age on the diagnostic yield, we stratified the diagnostic yields by age for both subgroups in our study, and found that the 2–5-year-old patients had the highest diagnostic yield of 3.5% (4/114) in ASD subgroup, while the highest yield of 40.3% (25/62) was observed in the 1–2-year-old cases in the ID/DD subgroup (Table 1). The 1–2-year-old ID/DD patients presented the most comorbidities for other abnormalities, such as cerebral palsy, facial dysmorphism, microcephalus, and epilepsy. The mean age of the subgroups in our study was significantly different (*P* = 1 × 10^−3^), being 4-year-1-month for ASD and 2-year-11-month for ID/DD, which may have contributed to the difference in yield between the two subgroups.

Aneuploidy is the gain or loss of an entire chromosome and is the leading genetic cause for developmental abnormality^26^. To better assess the advantages of CMA for identification of clinically relevant CNVs in ASD and ID/DD with clear phenotypes in northern China, the patients with aneuploidy such as trisomy 21, 47XXY, 47 XYY syndromes were excluded at first. In this study, 4 CNVs in ASD subgroup were associated with known chromosomal syndromes, including Kleefstra syndrome, *AUTS2* syndrome, 15q24 microdeletion syndrome, and *MECP2* duplication syndrome. In the ID/DD subgroup, 60 CNVs occurred at loci associated with 37 known chromosomal syndromes. Recurrent pathogenic CNVs were most frequently detected at 7q11.23, corresponding to the Williams–Beuren syndrome deletion which was observed in 11/251 (4.4%) ID/DD patients. Other chromosomal syndromes observed in multiple patients with ID/DD included partial trisomy 9p (duplication at chr9p24), 15q11q13 duplication syndrome, 18q deletion syndrome^34^, Prader–Willi syndrome, 22q11 deletion syndrome (DiGeorge syndrome), 8p23.1 microdeletion syndrome, 22q11.2 duplication syndrome, and *MECP2* duplication syndrome.

It has been recommended that variants of uncertain significance be considered in the diagnostic yield of ID/DD, particularly ASD, as many CNVs of VUS will change to pathogenic CNVs following the accumulation of clinical evidence in databases^31^. By analyzing CNVs interpreted as VUS in this cohort, the diagnostic yield of ASD subgroup increased from 3.3% (5/151) to 21.2% (32/151). The 27 CNVs interpreted as VUS in the ASD subgroup included 17 duplications with size of 56 kb to 1.7 Mb and 10 deletions with size of 74 kb to 1.2 Mb. These CNVs impacted some genes related to ASD/DD and were considered as new CNV candidates for association with ASD after analyzing data from databases and the literature. For example, a 141 kb duplication at 2q14.1 involving *DPP10* was detected in a 3-year-old autistic boy. *DPP10* has been reported to be related to synaptogenesis and ASD susceptibility in several studies of autism^35–37^. However, Mak et al. recently proposed that *DPP10* duplication is likely a benign CNV polymorphism enriched in Southern Chinese with a population frequency of ~1% by genotyping 258 Southern Chinese ASD patients^38^. In DECIPHER, a CNV of the same size was reported in a patient with autistic behavior and mild global developmental delay. More samples, especially Chinese from northern China, are needed to clarify the genotype-phenotype relationship of this CNV. A second example is a 477 kb duplication at 2q14.3 involving *CNTNAP5* that was detected in a 2-year-4-month girl. Rare deletion of *CNTNAP5* was suggested as a novel genetic factor that might confer ASD susceptibility^39^. However, duplications of *CNTNAP5* have not been studied. Last, a 115 kb maternal duplication at 2q36.3 was identified in a 2-year-4-month female autistic patient with ASD, and overlaps *TRIP12*, which has been reported to be associated with ASD^40^. An 180 kb duplication at the 5′ portion of *TRIP12* has been reported in an individual with macrocephaly^41,42^. Two additional duplications on 2q36.3 with similar sizes were found in two cases with ID and DD in the DECIPHER database.

In this study, we investigated the genetic etiology of a clinical cohort of NDD subjects who presented with ASD or ID/DD. The cohort was comprised of Han Chinese subjects from Shandong province, a northern region of China, who presented with typical phenotypes and were diagnosed by experienced pediatricians or pediatric neurologists. In our cohort, the patients with ID/DD were of a younger age than the patients with ASD. The high diagnostic yield observed in our cohort may have been influenced by the clinical experience of the referring pediatricians, the phenotypic severity of the cohort, gender, and age. Genome sequencing is expected to provide a higher yield on this clinical cohort^43–45^.

## Methods

### Ethics statement

The study was approved by the Ethics Committee of Qilu Children’s Hospital of Shandong University. Informed written consent was obtained from the patients’ parents. The information of the patients’ and their families was anonymized prior to genotyping and analysis. All the procedures performed in the study were in accordance with the Declaration of Helsinki.

### Subjects

A total of 410 probands with ASD or unexplained ID/DD with or without other congenital anomalies who were referred for genetic services in our institute from January 2014 to December 2018 were enrolled in this study. Their parents were also enrolled in the study. The cohort consisted of 282 male and 128 female patients (male:female ratio = 2.20) with mean age of 2-years-11-months, including 151 unrelated ASD patients (127 males and 24 females, ratio = 5.29) with mean age of 4-years-1-month (ranged from 1-year-10-months to 8-years-2-months) and 259 ID/DD patients (155 male and 104 female, ratio = 1.49) with mean age of 1-year-10-month (ranged from 1 day to 10-year-3-month) (Supplementary Data 1). There were 40 fathers and 24 mothers who were unavailable for testing. The 151 ASD patients were diagnosed by experienced pediatric neurologists at the Pediatric Health Care Institute using the criteria defined in the Diagnostic and Statistical Manual of Mental Disorders, 5th Edition (DMS-5) (American Psychiatric Association, 2013), the Autism Diagnostic Observation Scale (ADOS-2, 2002), and confirmed with the Children Autism Rating Scale (CARS, score >30). The 259 ID/DD patients were diagnosed by experienced pediatric neurologists at the Pediatric Health Care Institute, Rehabilitation Center and Neurology Department in Qilu Children’s Hospital of Shandong University according to the DMS-5 criteria, and the diagnosis was confirmed using the Gesell development scales with DQ < 75 and Wechsler Intelligence Scale for Children-Revised with IQ < 70. The exclusion criteria for this study were: (1) organic diseases of the nervous system, such as cerebral palsy, chronic epilepsy, encephalitis, meningitis, severe brain injury, brain surgery; (2) severe systemic physical diseases, such as those of the heart, liver, kidney, endocrine, and circulation; (3) Schizophrenia and other mental disorders, such as attention deficit hyperactivity disorder (ADHD) and obsessive compulsive disorder (OCD); (4) abnormal organic acids in blood and urine screening tests.

All participants in this study were ethnically and geographically homogenous Han Chinese recruited from Shandong province. Five subjects with highly suspected trisomy 21 syndrome were analyzed by G-banding karyotyping based on recommendation of the clinicians, and the remaining 405 patients were analyzed by CMA using the Affymetrix CytoScan HD array or Affymetrix Human Genome-Wide SNP 6.0 array.

### Genotyping analysis

Genomic DNA was extracted from peripheral blood samples of the probands and their parents using TIANamp Blood Genomic DNA Purification Kit (TIANGEN, Beijing, China) following the manufacturer’s instructions. Potential RNA contamination was removed by RNaseA (TIANGEN, Beijing, China). The DNA was quantified using the NanoDrop ND-1000 spectrophotometer (Thermo Fisher, Waltham, MA, USA). The genomic DNA was genotyped using the Affymetrix Human Genome-Wide SNP Array 6.0 or Affymetrix CytoScan HD Arrays (Affymetrix, Santa Clara, Calif., USA). DNA digestion, ligation, fragmentation, labeling, hybridization, staining and scanning were performed following the manufacturer’s protocols (Affymetrix, Santa Clara, CA).

### Data analysis and CNV evaluation

The data were analyzed with Command Console 3.1 (Affymetrix, Santa Clara, CA) or Chromosome Analysis Suite (ChAS) version 3.1.0.15 (Affymetrix, Santa Clara, Calif., USA). Data quality was evaluated with contrast quality control (CQC). The default CQC threshold (≥0.4) was used for analyzing each sample. Samples with a CQC < 0.4 were excluded from the study. The QC call rates of all the samples were greater than 96%. The reporting threshold was set at 50 kb (markers ≥ 20) for deletions and duplications.

To evaluate the pathogenic associations of CNVs, the Database of Genomic Variants (DGV, http://projects.tcag.ca/variation), CAG database (CAGdb, http://www.cagdb.org), University of California Santa Cruz Genome Browser (UCSC, http://genome.ucsc.edu), Online Mendelian Inheritance in Man (OMIM, http://www.omim.org), DECIPHER database (http://decipher.sanger.ac.uk), ISCA (https://www.iscaconsortium.org), and PubMed (http://www.ncbi.nlm.nih.gov/pubmed) were used. The control data of 1679 non-ASD Chinese subjects from multiple sources were used to distinguish rare copy number variations in our cohort. The control data included 919 samples from Singapore database^46^, 103 samples from the HapMap project^47^, 451 samples from Lu et al.^48^, and 206 parents from Gazzellone et al.^49^. The frequency of prioritized CNVs was computed against the aforementioned controls. CNVs with >50% reciprocal overlap were deemed identical^50^. Rare CNVs were defined as those not being present in more than 1% of 1679 ethnically-matched non-ASD control samples. We further restricted rare CNVs to those not being present in more than 1% of subjects in the Database of Genomic Variants (DGV)^51^. We analyzed CNVs overlapping <70% of their total length with segmental duplications and repeat-rich loci of the human genome. All CNVs were classified as pathogenic, likely pathogenic, variants of uncertain significance (VUS), likely benign, and benign according to the American College of Medical Genetics and Genomics guidelines^52^. In brief, CNVs were considered as pathogenic if they were documented as clinically significant in multiple peer-reviewed publications, or large CNVs unreported in the literature but overlapped a smaller interval with established clinical significance; CNVs were regarded as likely pathogenic if they were described in a single case report but with well-defined breakpoints and phenotype associated with NDDs, or involved a gene with a very compelling function-related and specific to NDDs. Other classes of CNVs were considered variants of uncertain clinical significance (VUS), likely benign or benign CNVs. Pathogenic, likely pathogenic, and VUS CNVs were considered to potentially affect gene function associated with the phenotypes of ID/DD or ASD in the study and were further validated by MLPA/qPCR.

### Multiplex ligation-dependent probe amplification (MLPA)/quantitative PCR (qPCR)

MS-MLPA was performed using SALSA MLPA kits ME028 and ME030 (MRC Holland, Amsterdam, Netherlands) to identify PWS/AS, and Silver–Russell syndrome, separately, according to the manufacturer’s instructions. The data were analyzed using Coffalyser software. qPCR with SYBR Green chemistry were utilized to verify the potentially clinically relevant CNVs in the cases and their parents. The primer sets for qPCR were designed to target different fragments within variant regions using an online primer designing tool—Primer 3 (http://primer3.ut.ee/)—and were synthesized by Shanghai Invitrogen Biotechnology Company (Shanghai, China) (Supplementary Data 5 and 6). Assays were carried out in accordance with manufacturer recommendations on the 7500 Fast Real-Time PCR system (Applied Biosystems, Foster City, California). The copy number variations were determined based on the ratio of target region copies to reference gene (*GAPDH*) copies in samples. Both male and female genomic DNA samples from unaffected pooled samples stored in our laboratory were used simultaneously as male and female control samples. Each qPCR was carried out in triplicate with the SYBR Premix Ex Taq II PCR reagent kit (TakaRa Bio, Dalian, China) following the manufacturer’s protocol.

### Statistical analysis

Statistical analysis was performed with SPSS 16.0 software. Two-sided Fisher’s test was used to test significance of CNVs in different groups. Results were considered statistically significant when the *P* value was <0.05 and the confidence interval was 95%.

### Reporting summary

Further information on research design is available in the Nature Research Reporting Summary linked to this article.

## Supplementary information

Supplementary Information

Reporting Summary

Supplementary Data 1

Supplementary Data 2

Supplementary Data 3

Supplementary Data 4

Supplementary Data 5

Supplementary Data 6

## Data Availability

CNVs data has been deposited at the European Genome-phenome Archive (EGA), which is hosted by the EBI and the CRG, under accession number EGAS00001005763. Further information about EGA can be found on http://ega-archive.org.
