# Supplementary material for: Chromosomal microarray analysis of 410 Han Chinese patients with autism spectrum disorder or unexplained intellectual disability and developmental delay
Source: NPJ Genom Med. 2022 Jan 12;7:1. doi: 10.1038/s41525-021-00271-z (PMC8755789; doi:10.1038/s41525-021-00271-z)
Supplement: Supplementary file 1 — Supplementary Information [file 41525_2021_271_MOESM1_ESM.pdf]

Captions of the supplementary data files

Supplementary Data 1: Demographic of the patients

Supplementary Data 2: High quality CNVs in the cohort

Supplementary Data 3: Clinically relevant CNVs in ASD cases

Supplementary Data 4: Clinically relevant CNVs in ID DD cases

Supplementary Data 5: Primers used for CNVs in ASD

Supplementary Data 6: Primers used for CNVs in ID DD
