# Supplementary material for: Chromosomal microarray analysis of 410 Han Chinese patients with autism spectrum disorder or unexplained intellectual disability and developmental delay
Source: NPJ Genom Med. 2022 Jan 12;7:1. doi: 10.1038/s41525-021-00271-z (PMC8755789; doi:10.1038/s41525-021-00271-z)
Supplement: Supplementary file 2 — Reporting Summary [file 41525_2021_271_MOESM2_ESM.pdf]

nature research

Corresponding author(s): Scherer SW & Gai Z

Last updated by author(s): Mar 25, 2021

Reporting Summary

Nature Research wishes to improve the reproducibility of the work that we publish. This form provides structure for consistency and transparency in reporting. For further information on Nature Research policies, see our Editorial Policies and the Editorial Policy Checklist.

Statistics

For all statistical analyses, confirm that the following items are present in the figure legend, table legend, main text, or Methods section.

n/a	Confirmed	
☐	☒	The exact sample size (n) for each experimental group/condition, given as a discrete number and unit of measurement
☐	☒	A statement on whether measurements were taken from distinct samples or whether the same sample was measured repeatedly
☐	☒	The statistical test(s) used AND whether they are one- or two-sided<br>*Only common tests should be described solely by name; describe more complex techniques in the Methods section.*
☒	☐	A description of all covariates tested
☒	☐	A description of any assumptions or corrections, such as tests of normality and adjustment for multiple comparisons
☒	☐	A full description of the statistical parameters including central tendency (e.g. means) or other basic estimates (e.g. regression coefficient) AND variation (e.g. standard deviation) or associated estimates of uncertainty (e.g. confidence intervals)
☒	☐	For null hypothesis testing, the test statistic (e.g. F, t, r) with confidence intervals, effect sizes, degrees of freedom and P value noted<br>*Give P values as exact values whenever suitable.*
☒	☐	For Bayesian analysis, information on the choice of priors and Markov chain Monte Carlo settings
☒	☐	For hierarchical and complex designs, identification of the appropriate level for tests and full reporting of outcomes
☒	☐	Estimates of effect sizes (e.g. Cohen's d, Pearson's r), indicating how they were calculated

Our web collection on statistics for biologists contains articles on many of the points above.

Software and code

Policy information about availability of computer code

Data collection	no software was used.
Data analysis	Command Console 3.1 or Chromosome Analysis Suite (ChAS) version 3.1.0.15 (Affymetrix, Santa Clara, Calif., USA) were used to analyze the data.

For manuscripts utilizing custom algorithms or software that are central to the research but not yet described in published literature, software must be made available to editors and reviewers. We strongly encourage code deposition in a community repository (e.g. GitHub). See the Nature Research guidelines for submitting code & software for further information.

Data

Policy information about availability of data

All manuscripts must include a data availability statement. This statement should provide the following information, where applicable:

- Accession codes, unique identifiers, or web links for publicly available datasets
- A list of figures that have associated raw data
- A description of any restrictions on data availability

The Genome-Wide SNP array and the CytoScan HD Array data used to support the findings of this study have been deposited in the public repository, and the accession code is 10.6084/m9.figshare.14301476.

Field-specific reporting

Please select the one below that is the best fit for your research. If you are not sure, read the appropriate sections before making your selection.

☒ Life sciences ☐ Behavioural & social sciences ☐ Ecological, evolutionary & environmental sciences

For a reference copy of the document with all sections, see nature.com/documents/nr-reporting-summary-flat.pdf

Life sciences study design

All studies must disclose on these points even when the disclosure is negative.

Sample size	Sample sizes were chosen with reference to similar literature in this field.
Data exclusions	Data exclusions were according to the manufacturer's instructions: Data quality was evaluated with contrast quality control (CQC). The default CQC threshold (≥0.4) was used for analyzing each sample. Samples with a CQC <0.4 were excluded from the study. The QC call rates of all the samples were greater than 96%. The reporting threshold was set at 50 kb (markers ≥20) for deletions and duplications.
Replication	qPCR with SYBR Green chemistry were utilized to verify the potentially clinically relevant CNVs. We selected 162 rare CNVs for experimental validation, of which 150/162 were confirmed as true calls.
Randomization	The cohort consisted of 151 ASD patients and 259 ID/DD patients which were diagnosed by experienced pediatricians or pediatric neurologists.
Blinding	The investigators were blinked to group allocation during data collection.

Reporting for specific materials, systems and methods

We require information from authors about some types of materials, experimental systems and methods used in many studies. Here, indicate whether each material, system or method listed is relevant to your study. If you are not sure if a list item applies to your research, read the appropriate section before selecting a response.

Materials & experimental systems

n/a	Involved in the study	
☒	☐	Antibodies
☒	☐	Eukaryotic cell lines
☒	☐	Palaeontology and archaeology
☒	☐	Animals and other organisms
☐	☒	Human research participants
☒	☐	Clinical data
☒	☐	Dual use research of concern

Methods

n/a	Involved in the study	
☒	☐	ChIP-seq
☒	☐	Flow cytometry
☒	☐	MRI-based neuroimaging

Human research participants

Policy information about studies involving human research participants

Population characteristics	The human research participants of this study were the patients with clinical diagnosis of ASD, or DD/ID.
Recruitment	A total of 410 patients with clinically diagnosed ASD or ID/DD referred to our institute for genetic services were recruited in this study.
Ethics oversight	The study was approved by the Ethics Committee of Qilu Children's Hospital of Shandong University.

Note that full information on the approval of the study protocol must also be provided in the manuscript.
